# Health information management students’ work-integrated learning (professional practice placements): Where do they go and what do they do?

**DOI:** 10.1177/18333583241303771

**Published:** 2024-12-18

**Authors:** Kerin Robinson, Merilyn Riley, Natasha Prasad, Abbey Nexhip

**Affiliations:** 1 La Trobe University, Australia; 2 Goulburn Valley Health, Australia

**Keywords:** health information management, health information manager, health information management profession, health information management workforce, work-integrated learning, higher education, preceptorship, experiential learning, allied health occupations, project-based placements, professional practice

## Abstract

**Background:** Work-integrated learning (WIL) is integral to most health disciplines’ profession-qualifying degree programs. **Objectives:** To analyse the categories, locales and foci of final-year (capstone), health information management professional practice (WIL) placements, 2012–2021, at La Trobe University, Australia. **Method:** A documentary analysis of 614 placement agency proposals, 2012–2021, interrogated multiple characteristics: agency type, placement (sub-) category (WIL model), project type, agency-required student capabilities, intended learning outcomes. **Results:** Public hospitals offered 50% of all placements. Medical research/health or disease screening/clinical registries offered 17.8%, incorporating 86.7% of “research-based” placements. Government department offerings were consistently stable; private hospital, primary care and community healthcare offerings declined. The majority (64.8%) of offerings were “project-based,” followed by “internship” (28.7%: Health Information Service (14%) and “other” (14.7%)), research-based (4.9%) and other (1.6%). Ninety-nine (16.1%) proposals specified additional, pre-placement skills and capabilities: technical (information technologies, software applications; 58.6% of 99 proposals); working independently (49.5%); communications (written, verbal; 45.5%); targeted interest (38.4%) in “informatics and data quality,” “quality and safety,” “software development,” “coding”; organisational and/or time management skills (29.9%); teamwork skills (20.2%); data analysis skills (18.2%); enthusiasm and/or self-motivation (15.2%). **Conclusion:** The project-based model for the capstone placement is ideal for preparing health information management students for complex, graduate professional work. Agencies’ pre-placement expectations of students (knowledge, technical skills, soft skills) are consistent with findings from the WIL literature and align with course curricula and Australia’s Health Information Manager (HIM) Profession-entry Competency Standards. **Implications:** The findings will strengthen the health information management profession’s knowledge base of WIL and inform educators, students and agency supervisors.

## Introduction

Australia’s Tertiary Education Quality and Standards Authority (Australian Government, Tertiary Education Quality and Standards Agency (TEQSA), 2022: 1) has defined work-integrated learning (WIL) as encompassing “any arrangement where students undertake learning in a work context as part of their course requirements.” These arrangements include professional placements, clinical placements, internships, workplace projects, supervised practice and simulations, and fieldwork (Smith, 2012; Universities Australia, 2021). The permeation of WIL into the core pedagogy of universities in Australia and other countries, as part of overarching strategies to produce competently skilled, work-ready graduates (Billett, 2009; Lester and Costley, 2010; Universities Australia, 2021), has amplified the need to explore its benefits. The concept of WIL draws upon long-standing theoretical underpinnings. These include Dewey’s (1938) learning-by-doing to achieve a mutually reinforcing industry-classroom education experience that also involves student reflection upon the activity to solidify and articulate knowledge, and Kolb’s (1984) experiential learning theories which emphasise that a student’s learning is enriched through “doing” the relevant activity.

Consistent with the long traditions of clinical and professional practice embedded in most health disciplines’ profession-entry university degrees, the health disciplines in Australia’s universities have the highest WIL participation rate of all disciplines (Universities Australia, 2021: 13). Within the health disciplines, WIL can be compartmentalised into various preceptorship arrangements. Five uniquely characterised placement models and approaches to supervision in the health disciplines have been posited in Australia (ClinEdAus, 2024): traditional; collaborative; multiple mentoring; role emerging; and project based.

### WIL models

Owing to the wide variation in WIL models, it is infeasible to nominate just one of these to evaluate the efficacy of WIL in meeting student placement objectives. Several underlying principles are consistent across WIL formats. Smith (2012: 247), in Australia, identified five key dimensions: “authenticity, integrated learning supports, . . . alignment (of teaching and learning activities and assessment with integrative learning outcomes), supervisor access and induction/preparation processes.” In developing a “proposed global WIL framework,” McRae and Johnston (2016: 341), in Canada, highlighted the importance of “experience, curriculum integration, student outcomes and reflection.” More recently, in Australia, Campbell et al. (2019) developed a framework to guide evaluation of WIL at the institutional level; this involves the four domains of student experience, curriculum design, institutional requirements and stakeholder engagement. Each of these three frameworks embodies the expectation that the WIL activities and experience are authentic and aligned with intended learning outcomes (ILOs). The two most commonly used WIL models in health information management courses are the project placement and academic internship models.

#### Project placement model

Project-based learning (PjBL) is a long-established learner-centred approach that falls under the umbrella of problem-based learning (Lee et al., 2014; Savery, 2006). In their study of Australian occupational therapy students’ placements, Fortune and McKinstry (2012) described the project placement as a “capstone” (p. 265) experience wherein the student applies a range of skills such as real-world problem solving, critical thinking and teamwork to reinforce and consolidate their profession-entry generic and discipline-specific competencies. The project-based capstone placement requires an organised orientation program and the explication of clear expectations of what is required by the student; these are reinforced by a welcoming learning environment that, as identified by Rodger et al. (2011), is applicable to all WIL models and lends itself to honest supervisor-student discussions. There is considerable evidence, from various professional disciplines in Australia and elsewhere, that enablers of an effective project placement include critical reflective practice to underpin continuing professional development (Fortune and McKinstry, 2012; Pegg et al., 2012; Thompson, 2016). A balance between autonomy and supervision is necessary: more extensive supervision is provided in the initial stages while students are developing the requisite skills to engage independently in their project, supported by clearly defined learning objectives, goals and actions to achieve these (Prigg and MacKenzie, 2002). Mate et al. (2024) found that constructive, balanced and, effectively, 360° feedback is essential. The afore-mentioned structures need to be underpinned by adequate support and regular communication from the university with both students and supervisors, provision of training tools to assist placement agencies in undertaking the supervision and availability of university supervisors to assist students with potential challenges (Fortune and McKinstry, 2012; Prigg and MacKenzie, 2002).

#### Academic internship model

Internships and project-based placements have differing aims. Typically, “academic” internships, sometimes called “professional” internships (King and Sweitzer, 2014), are traditionally a mandatory component of the professional degree. They are designed to provide important experiential education in which students undertake supervised, applied learning within a specific industry or field of study. In the health information management degrees, students can apply the theory-based knowledge gained during their academic studies and engage in the day-to-day professional activities undertaken by Health Information Managers (HIMs) in a range of workplace settings, thereby learning and acculturating to the profession’s “norms and values” (King and Sweitzer, 2014: 40). They gain valuable exposure to the real world of health information management, and the opportunity to assess whether this is the type of area or agency in which they might pursue their future career.

### Context

Experiential learning, by way of “professional” or “clinical” placements, has been integral to health information management education in Australia since the first formal programs commenced in the states of New South Wales in 1956 (Watson, 2013) and Victoria in 1961 (Ell, 1984). As exemplified by Marks and McIntosh (2006), the experiential learning sites used by courses externally accredited as being compliant with the profession-entry competency standards of the Health Information Management Association of Australia (HIMAA, 2017, 2023) have historically included hospitals, community healthcare agencies, research centres and government Departments of Health.

At Victoria’s La Trobe University (LTU), the face-to-face and online profession-entry health information management programs have embodied the afore-mentioned models at different times. A traditional or collaborative model is applied in the first and second years of the undergraduate degree and the first year of the postgraduate, qualifying degree. In the final-year placement for both programs, students engage in a project, role emerging or traditional model of WIL. The models were adopted intentionally to expose students to a range of learning experiences in preparation for a straightforward transition to their future professional workplaces. Geographically, LTU’s placements are undertaken in all Australian states and territories. A planned course restructure at LTU has foreshadowed a shift of final-year placements to the project model.

#### Aims

There is negligible published research on WIL in health information management university programs. This study was part of a larger project that aimed to evaluate LTU’s health information management professional practice placement program and identify evidence to inform best practice guidelines for project-based placements. The objective of the component of the study reported herein was to analyse the categories, locales and foci of final-year (capstone) health information management professional practice (WIL) placements, 2012–2021, at LTU.

## Method

### Study design

A qualitative research method, documentary analysis informed by Morgan (2021), Dalglish et al. (2020), Gorsky and Mold (2019) and Bowen (2009), was utilised to obtain data on previous student placements. This unobtrusive method was chosen because of the ready availability and inherent stability of the data, time- and cost-effectiveness of the data collection, and minimal ethical issues associated with the use of extant documents (Morgan, 2021).

### Sample

A purposive sampling approach was used to identify eligible documents. The documents that constituted the sample comprised the professional practice placement proposals for all final year placements (*n *=* *643) undertaken in LTU’s health information management degrees from 2012 to 2021, inclusive. The placement proposal is created by the placement agency. It is the core, underpinning document that articulates and shapes the parameters and scope of the placement, from the perspective of the host agency. Each proposal had been provided to the university several months prior to the respective placement. The proposals outlined the nature and scope of the activities to be undertaken by the students; they were absent student identification details. An exclusion criterion was applied in cases where no placement proposal could be readily located or where there were duplicate proposals in any year. This resulted in the final sample of 614 proposals.

A pilot study was undertaken using a randomly selected sub-sample of project placement templates from 2010 because of course changes in 2011 and to guide refinement of classification of placement proposal categories.

### Materials and procedure

#### Data collection tool

An Excel template, incorporating the variables shown in Box 1, was developed to extract the data items from the placement proposals.

**Box 1.** Data collection items.

Data item collected	Format	
Proposal identifier	Number	
Year of placement	Year	
Presence of organisational overview	Yes/No	
Placement agency type	Select: • Public hospital or network • Private hospital or group • Government department • Health-related IT firm • Medical/health research or disease screening organisation	• Not-for-profit health consumer organisation • Primary care or community healthcare agency • Health insurance fund • Other
Provision of placement details	Yes/No	
Placement category	Select: • Internship (HIS) • Internship (Other) • Project-based (HIS) • Project-based (IT)	• Project-based (Allied Health) • Project-based (Other) • Research-based • Other • Not specified
Major or minor project (Definitions in Supplemental Appendix A)	Select: • Major • Minor	• Major or Minor • Not applicable • Unknown/not specified
Placement sub-category (Definitions in Supplemental Appendix A)	Select: • Hospital-based HIS • Informatics or Health IT • Epidemiological Research or Medical Research • Quality or Clinical Risk Management • Clinical Coding and/or Casemix • HIS Special Project • Mixed options	• Disease/Screening Registry or Clinical Database Management • Health Data Analysis and/or reporting • State or national information infrastructure and government • Process, policy and/or procedure development • Not specified
Further project details	Free text	
Expectations of student capabilities prior to placement are expressed on the form	Yes/No	
Specified learning outcomes	Select up to six: • Communication skills (written (including report writing) and/or verbal) • Computer skills (e.g. MS Word, Excel) • Technical skills (statistical software packages, software applications) • Project management skills • Ability to work independently • Data analysis and reporting skills • Policy and/or procedure development • Clinical coding skills • Knowledge in quality improvement activities • Time management • Interpersonal skills • Attention to detail • Teamwork • Research skills • Experience in health information management skills • Flexibility • Data quality skills • Free text (if not above)	
Formal template completed	Yes/No	

HIS: Health Information Service.

#### Documentary analysis

Content analysis was undertaken for this component of the study. An iterative process of skimming, reading and interpretation was applied. Box 2 shows the definitions of placement models utilised to assign the placement proposal categories.

**Box 2.** Definition of broad placement categories.

*Internship placement*. Involves participation in the day-to-day management activities of the host organisation, for example, hospital Health Information Service, clinical quality registry, research centre. This placement allows the student to interact with a wide range of staff members and engage in multiple “routine” health data- and information-related functions across the healthcare setting. *Project placement*. Per Prigg and Mackenzie’s definition (2002: 213): “. . . students complete a piece of work initiated by a fieldwork site . . . under the supervision or an appropriately qualified supervisor.” Key features of this model include self-direction, student negotiated objectives and minimal supervision. *Research placement*. A research-based placement (involving any stage of a study). *Other placements*. Residual category: caters for placements that do not meet the definition of an internship, project placement or research placement.

#### Data extraction

Following the pilot study, a data dictionary and internal rules (Supplemental Appendix B) were established, based on mutual agreement by the four researchers (N.P., K.R., M.R., A.N.). These were refined iteratively. Each proposal was independently assessed by a pair of the researchers (N.P., K.R., M.R., A.N.) to classify multiple variables. Discrepancies identified by each pair were addressed at regular, all-reviewer team meetings and issues were resolved by consensus. In order to minimise potential researcher-bias, paired researchers independently reviewed all project proposals and assigned the categories, prior to a consensus approach involving the full research team.

#### Data analysis

Descriptive statistics were utilised to provide frequency tables that reported on several variables.

### Ethics approval

Ethics approval for the project was granted by the LTU Human Research Ethics Committee (HREC; No. HEC22060).

## Results

A total of 614 placement proposals were retrieved for study following exclusion of 29 missing or duplicate proposals.

### Placement categories

“Project-based placements” constituted the majority (64.8%) of all health information management placements offered during the study timeframe. These were followed by “internships” (*n *=* *176, 28.7%), “research-based” placements (*n *=* *30, 4.9%) and those classified as “other” (*n *=* *10, 1.6%) per the definitions shown in Box 2. Placements assigned to the latter category included those for which no placement details were provided in the proposal or where multiple, mixed options (e.g. internship with a major project) were offered.

**Figure 1.**
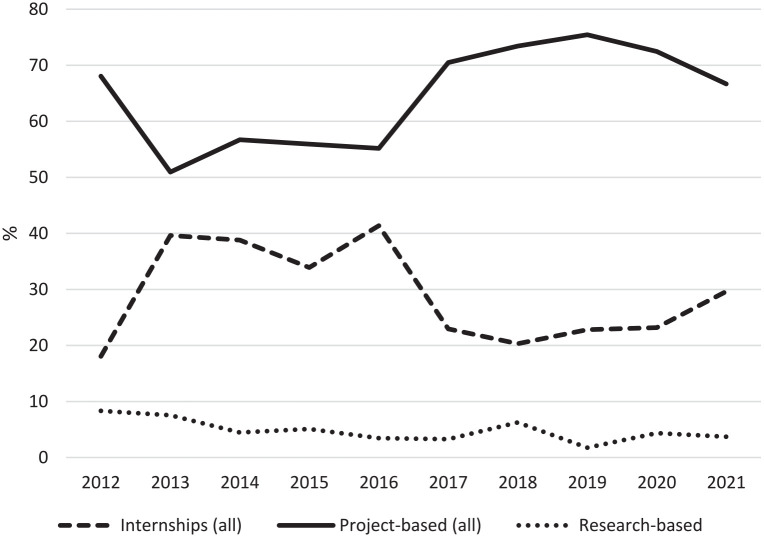
Trends in broad categories of placements (internships, projects, research), 2012–2021. *Note*: The “other” category has been excluded from this graph due to small figures and instances being offered only intermittently during the study period.

The annual proportions of internships and project-based placements fluctuated over the study period (Figure 1); for instance, the 2017–2019 triennium saw an increase in project-based placements and a corresponding decrease in internships. Research-based placements, the smallest in number, demonstrated a slow decline from 2012. Table 1 shows the relative proportions of all placement categories, with further division into hospital Health Information Service (HIS)-based internships and other internships, and the broad area within the host agency of proposed project placements.

**Table 1.** Placement as broad category and sub-categories (internship, project-based, research-based, other) by year, 2012–2021.

Year	2012	2013	2014	2015	2016	2017	2018	2019	2020	2021	Total
*n*	72	53	67	59	58	61	64	57	69	54	614
	Column percent (%)										
Internships											
HIS Internship	11.1	20.8	22.4	16.9	17.2	6.6	10.9	7.0	7.2	22.2	14.0
Internship (Other)	6.9	18.9	16.4	16.9	24.1	16.4	9.4	15.8	15.9	7.4	14.7
Total	18.0	39.7	38.8	33.9	41.3	23.0	20.3	22.8	23.1	29.6	28.7
Project-based											
Project-based (HIS)	30.6	22.6	20.9	18.6	15.5	18.0	29.7	22.8	24.6	18.5	22.5
Project-based (IT)	12.5	9.4	9.0	5.1	13.8	6.6	7.8	3.5	13.0	9.3	9.1
Project-based (Allied Health)	0.0	0.0	0.0	0.0	6.9	8.2	20.3	21.1	13.0	16.7	8.5
Project-based (Other)	25.0	18.9	26.9	32.2	19.0	37.7	15.6	28.1	21.7	22.2	24.8
Total	68.1	50.9	56.8	55.9	55.2	70.5	73.4	75.5	72.3	66.7	64.9
Research-based	8.3	7.5	4.5	5.1	3.4	3.3	6.3	1.8	4.3	3.7	4.9
Other/Not specified	5.6	1.9	0.0	5.1	0.0	3.3	0.0	0.0	0.0	0.0	1.6
Total	100.0^a^	100.0^a^	100.0^a^	100.0	100.0^a^	100.0	100.0	100.0^a^	100.0^a^	100.0	100.0^a^

HIS: Health Information Service.

a Rounding applied.

#### Internships

There were relatively equal proportions, of all placements offered, of HIS-based internships (14.0%) and all other internships (14.7%) (Table 1). The annual proportion of HIS-based internships varied from 22.4% (2014) to 6.6% (2017) (Table 1). The proportion of other types of proposed internships also fluctuated, from 6.9% (2012) to 24.1% (2016): these incorporated day-to-day health data- and information-related management activities in government departments, clinical quality registries and specialist organisations including cancer registries and epidemiology centres.

#### Project-based placements

The “other” category represented the highest proportion of project-based placements (24.8%); the greatest proportion, observed in 2017 (37.7% of all placements), declined by 22.1% in 2018 (to 15.6% of all placements). This category included projects in organisations such as public hospital quality/clinical governance departments, disease registries, community healthcare services and government departments. Next, there was a higher proportion of HIS project-based placements (22.5%) compared to information technology-focused (9.1%) and allied health-based project placements (8.5%). Allied health project-based placements were first offered in 2016 and reached their highest proportion in 2019 (21.1%). Project-based HIS placements were available consistently throughout the study period, with the lowest proportion occurring in 2016 (15.5%). Notably, five of the project-based HIS proposals were from primary care or community healthcare agencies: the patient-record based nature of these projects reflected work typically performed in a hospital HIS and this category most accurately reflected the nature of the projects.

Project-based placements (*n *=* *398) were also classified into major (*n *=* *254, 63.8%), major or minor (*n *=* *103, 25.9%), minor (*n *=* *29, 7.3%) or unknown/not specified (*n *=* *12, 3.0%).

### Placement agency type

Table 2 shows the proportions of all placements, according to agency type and year. Public hospitals accounted for 50% of all placement proposals and were significantly more likely than other agency types to offer placements. Whilst the collective category of medical research/health or disease screening/clinical (quality) registry represented significantly fewer placements than public hospitals, fluctuating over the study period, they constituted the second highest source of final year health information management placements (average of 17.8%). Government departments and private hospitals also demonstrated steady proportions of placements offered, although an overall decline was observed in the latter. The “other” placement agency type comprised a small, fluctuating number of placements. The most obvious decline was observed in placements offered by primary care and community healthcare agencies.

**Table 2.** Proportion of placement agency types, 2012–2021.

Placement sub-category (nature of the placement)	2012	2013	2014	2015	2016	2017	2018	2019	2020	2021	Total
*n*	72	53	67	59	58	61	64	57	69	54	614
	Column percent (%)										
Public hospital/network	48.6	45.3	47.8	49.2	34.5	54.1	57.8	50.9	47.8	64.8	50.0
Medical research/health or disease screening/registry	11.1	18.9	14.9	16.9	12.1	26.2	12.5	22.8	21.7	22.2	17.8
Government department	12.5	7.5	10.4	10.2	22.4	6.6	10.9	17.5	13.0	7.4	11.9
Private hospital/group	9.7	9.4	10.4	10.2	15.5	4.9	10.9	7.0	4.3	0.0	8.3
Primary care and community healthcare agencies	12.5	9.4	9.0	1.7	5.2	3.3	1.6	0.0	4.3	5.6	5.4
Not-for-profit health/consumer organisation	1.4	1.9	3.0	3.4	3.4	3.3	4.7	1.8	1.4	0.0	2.4
Health insurance fund	0.0	0.0	1.5	3.4	3.4	1.6	1.6	0.0	0.0	0.0	1.1
Health-related IT firm	2.8	7.5	1.5	1.7	1.7	0.0	0.0	0.0	2.9	0.0	1.8
Other	1.4	0.0	1.5	3.4	1.7	0.0	0.0	0.0	4.3	0.0	1.3
Total	100.0	100.0^a^	100.0	100.0^a^	100.0^a^	100.0	100.0	100.0	100.0^a^	100.0	100.0

a Rounding applied.

Almost 65% of all placements offered were project-based. Table 3 shows that the highest proportions of placement offerings from primary care and community healthcare services (87.9%) and public hospitals (72.6%) were project-based; a quarter of all public hospital-based placements offered were internships. Over half of all placements offered by several placement agency types were project-based; the exceptions were health-related IT firms, health insurance firms and medical research/health or disease screening/registry sites that each offered project-based placements in ⩽45.5% of cases. Over 64% of the placements offered in medical research/health or disease screening/registry agencies were either project-based or research-based. Medical research/health or disease screening/registry categories offered 86.7% of all research-based placements.

**Table 3.** Proportion of broad placement category by placement agency type.

Placement agency type	Internship	Project-based	Research	Other	Not specified	Total (*n*)
Row percent (%)						
Public hospital/network	25.4	72.6	0.0	1.3	0.7	307.0
Medical research/health or disease screening/registry	34.9	41.3	23.9	0.0	0.0	109.0
Government department	26.0	69.9	4.1	0.0	0.0	73.0
Private hospital/group	37.3	56.9	0.0	3.9	2.0	51.0
Primary care and community healthcare agencies	9.1	87.9	0.0	0.0	3.0	33.0
Not-for-profit health/consumer organisation	40.0	60.0	0.0	0.0	0.0	15.0
Health-related IT firm	54.5	45.5	0.0	0.0	0.0	11.0
Health insurance fund	57.1	42.9	0.0	0.0	0.0	7.0
Other	37.5	50.0	12.5	0.0	0.0	8.0
Total	28.7	64.8	4.9	1.0	0.7	614.0

### Placement foci

Table 4 provides details of the year and nature (foci) of the placements. The highest proportion of placements, overall, reflected the “Health Data Analysis and/or reporting” sub-category (15.6%); this statistic fluctuated before increasing steadily from 2019. Offerings in the “HIS Special Project” category constituted the next highest proportion (13.0%), remaining mostly consistent throughout the study timeframe with reductions in 2014 and 2016. Whilst these sub-categories accounted for the highest number of placements offered, the distribution among all sub-categories was relatively evenly balanced.

**Table 4.** Proportion of placement subcategories (nature of placement)^a^ by year, 2012–2021.

Placement sub-category (nature of the placement)	2012	2013	2014	2015^b^	2016	2017	2018	2019	2020	2021	Total
*n*	72	53	67	59	58	61	64	57	69	54	614
	Column percent (%)										
Health Data Analysis and/or reporting	11.1	1.9	13.4	11.9	6.9	11.5	21.9	21.1	24.6	31.5	15.6
HIS Special Project	16.7	15.1	4.5	16.9	8.6	9.8	17.2	10.5	17.4	13.0	13.0
Informatics/Health IT	9.7	13.2	11.9	13.5	20.7	8.2	14.1	8.8	17.4	9.3	12.7
Hospital-based HIS internship	9.7	20.8	16.4	13.5	10.3	3.3	6.3	5.3	5.8	20.4	10.9
Mixed options	8.3	9.4	13.4	13.5	13.8	13.1	6.3	1.8	10.1	7.4	9.8
Quality or Clinical Risk Management	9.7	9.4	7.5	8.5	6.9	16.4	10.9	12.3	4.3	3.7	9.0
Process, policy and/or procedure development/monitoring	8.3	7.6	11.9	3.4	6.9	8.2	0.0	8.8	4.3	0.0	6.1
Clinical Coding and/or Casemix	8.3	3.8	7.5	5.0	5.2	4.9	7.8	7.0	5.8	3.7	6.0
Epidemiological Research or Medical Research or Clinical Trials	5.6	7.5	7.5	5.0	5.2	6.6	7.8	5.3	1.4	3.7	5.5
State/national information infrastructure and governance	6.9	5.7	4.5	3.4	12.1	6.6	3.1	10.5	2.9	3.7	5.9
Disease or Screening Registry or Clinical Database Management	1.4	3.8	1.5	1.7	3.4	11.5	4.7	8.8	5.8	3.7	4.6
Not specified	4.2	1.9	0.0	5.0	0.0	0.0	0.0	0.0	0.0	0.0	1.1

HIS: Health Information Service.

a See Supplemental Appendix A for the definitions of these placement sub-categories.

b Rounding applied to this column.

### Outputs

The proposed WIL outputs comprised reports, presentations, educational materials, audits, system implementations, literature reviews, journal articles for peer-review, development and conduct of surveys, investigation into specialised classification systems, classification of diseases and more.

### Prior capabilities

Ninety-nine (16.1%) of the 614 proposals under study specified skills and capabilities required of the student by the placement agency, prior to placement. The most commonly required capability was skill in basic information and communications technologies applications (*n *=* *58, 58.6%) involving competent use of the Microsoft Office Suite including Access, Excel and Word, and knowledge of the reference management software, EndNote. The second most commonly required capability was the ability to work independently (*n *=* *49, 49.5%). Communication skills (written and verbal) were requirements listed in 45 (45.5%) of the sub-group of 99 proposals: the expected capability specified “strong,” “excellent,” “good” and “well-developed” communication skills. The fourth most commonly listed requirement was interest in a specific knowledge area (*n *=* *38, 38.4% of the 99 proposals), including interest in “informatics and data quality,” “quality and safety,” “software development” or “coding.” Twenty-nine proposals (29.3% of this sub-group) specified organisational and/or time management skills. Other required capabilities included the ability to work in a team (*n *=* *20, 20.2%), skills in data analysis (*n *=* *18, 18.2%) and enthusiasm and/or self-motivation (*n *=* *15, 15.2% of the 99 proposals).

## Discussion

The objectives of this component of the study, which involved the comprehensive analysis of 10 years of WIL capstone placement proposals, were to analyse the final year (capstone) professional practice placement (WIL) categories, locales and foci for the decade 2012–2021. The analysis revealed variability of the types of agencies that offered placements. This finding was reflective of historical practice in externally (HIMAA) accredited health information management degrees in Australia, although the wider range of agency types represented in the current study was arguably consistent with contemporary health information management practice. The placement activities undertaken by students were also consistent with the range of HIMAA-specified profession-entry competencies required of graduates completing HIMAA-externally accredited health information management courses.

### Internships versus project-based placements

The LTU final-year placements have traditionally embodied both the academic internship and project-based placement models. Internship placements, involving engagement in the daily health information management activities of the host agency, were historically linked closely to hospital HISs. In 2010, LTU’s academic staff created a generic outline of student activities in an HIS internship placement to accommodate some agencies that wished to offer placements, but found it difficult to devise a specific project. This was distributed to agencies with the advisory that where an agency was unable to create a project, an internship placement comprising smaller projects and day-to-day activities would constitute an alternative. The findings indicate that over successive years more internship-based placements were also offered in non-hospital/non-HIS settings such as registries, disease screening centres and government departments. The variety of settings in which internships were offered suggests that they are popular options for agencies involved in the final-year (capstone) health information management WIL experience.

LTU has generally aimed to source project placements rather than internships for its final-year health information management students. Placements that involve PjBL are, according to Australian research by Brewer et al. (2022), essential in preparing students, including those undertaking health courses, for “a disrupted employment market that requires new and diverse graduate capabilities” (p. 17). Project-based placements offer students “particularly rich learning opportunities” (Fortune et al., 2013: 219) whilst they engage in the construction of knowledge (Guo et al., 2020) to solve real-world problems. The students practice what Lee et al. (2014: 20) described as “21st century skills” including “collaboration, communication and critical thinking” to create “high-quality, authentic products and presentations” (p. 20). Projects enable students to focus on and achieve a particular outcome or deliverable, thereby affording them the opportunity to contribute significantly to their placement agency’s strategic goals or outcomes. These attributes suggest that the project-based placement model is ideal for the capstone professional practice placement in health information management degrees: the students are being prepared for future complex professional work that is centred in workplaces that are undergoing constant change in digitisation, technologisation and practices in the innovative performance of managing highly sensitive and critically important health data and information.

#### Major and minor projects

The categorisation of project-based placements into “major,” “major or minor” or “minor” proved definitionally challenging in the absence of an extant definition. The creation of internal definitions (see Supplemental Appendix A) revealed some inherent subjectivity in interpreting what may constitute a “substantial” output or body of work.

#### Placement agency types

Traditionally, the role of HIM was dispersed across the healthcare system, but predominately located within public or private hospital HISs (Watson, 2008). This pattern remains: Gjorgioski et al. (2023) found that 68.5% of LTU graduate HIMs from 2017 to 2021 were employed in their first position in public hospitals, 11.5% in private hospitals, 9.2% in research/cancer/screening or registry roles and 5.4% in government departments; that is, approximately 30% of new graduates undertook non-hospital roles. Public hospitals remain the major employer of new graduate HIMs. This is broadly consistent with the number and types of placements offered by agencies, as demonstrated in our analysis of the agency types. The graduate employment rate in public hospitals (68.5%) in 2017–2021, however, exceeded the proportion of student placements (50%) offered by public hospitals. This implies that either other agency types that offer placements do not have opportune vacancies, the non-public hospital sector tends to employ more experienced HIM graduates, or graduating students may have already accepted roles in public hospitals thereby making them unavailable to other potential employers at the time of final-year placement.

#### Trends in placements 2012–2021

Our findings demonstrate that final-year placement numbers fluctuated. Trends were not significant as the number of placements sought and/or sourced usually reflects the size of the relevant student cohort; anecdotally, in most years the number of placement proposals received exceeds the number of eligible students.

The decline in 2020 and 2021 in project-based placement offerings and the corresponding increase in internships is likely to have reflected the transition of most placements to off-site (online) mode owing to restrictions associated with the COVID-19 pandemic. This mode of placement delivery was consistent with courses in different disciplines and other countries (e.g. Bawadi et al., 2023; Martin et al., 2022; Teng et al., 2022). Difficulties were experienced by LTU in sourcing some types of placements because restrictions applied nation-wide and were especially onerous in the state of Victoria (Toole and Crabb, 2022; Vally and Bennett, 2021).

### Nature and foci of placements

The placement foci demonstrated the range and variability of the placements offered. These characteristics reflect the breadth of skill domains that students learn from the university curriculum and, also, provide them the opportunity to engage in placements suited to their professional interest. Riley et al. (2020) and Gjorgioski et al. (2023) reported that most LTU health information management graduates from 2012–2016 to 2017–2021, respectively, used three or more of the four, core professional knowledge-skill domains (i.e. data management and analytics, health classification, health information systems foci and health information management) in their first position.

Placement agencies’ increasing expectations that students bring health data analysis skills to placement activities reflects industry expectations for HIMs to manage and interpret the markedly increased volume of data now produced through technological advancements including electronic health records, big data analytics and generative artificial intelligence (Gjorgioski et al., 2023; Šendelj, 2020). Notably, only 6% of all project-based placements accounted for the highly specialised clinical coding/health classification domain; however, it is important to note that HIS internships often include some clinical coding (Table 4).

### Pre-placement requirements

Our analysis of the skills and capabilities required of students by some placement agency supervisors revealed expectations of a combination of professional and technical “hard” skill-sets and “soft” skills. There is a body of evidence that WIL supports students’ work readiness (Dolce et al., 2020; Ho, 2023; Jaaffar et al., 2016) and provides graduates with a “strong labour market advantage” (e.g. Jackson and Rowe, 2023: 490). The agency supervisors’ expectations of students’ competence in the use of various technologies, and their possession of soft skills such as teamwork, effective communication and time management, are potentially valuable in informing both students and the curriculum. Soft skills positively affect work productivity (Hamid and Younus, 2022). The agencies are potential employers of health information management graduates and their demand for soft skills aligns with the findings of Börner et al. (2018) which revealed their “critical importance” (p. 12631) in the workplace and the phenomenon that increasing demand for hard technical skills “often stimulates subsequent demand” (p. 12631) for soft skills.

### Limitations

A limitation of the study concerned the quality of certain placement proposals as some demonstrated paucity of both volume and quality of content, thereby limiting the amount of extractable data. (This problem will be reported separately.) Furthermore, the proposal template had been amended in 2012; consequently, placement agencies had less familiarity with the required template in the early years of the study. Other limitations concerned definitional challenges relating to an internship and a project-based placement, and the fact that only 16% of the proposals stipulated agencies’ pre-placement expectations of students. Finally, placement proposals did not always reflect what the student ended up doing. The proposals were necessarily requested by the university several months prior to the placement and unforeseeable agency constraints such as changes in staffing, resource availability, space and other factors, could necessitate a modification of student placement activity; such changes were not captured in the analysis.

## Conclusion

According to ten Cate (2021), the well-established scholarly tradition of medical education is transitioning to a broader domain of scholarship that incorporates other health professions. There is a relatively meagre record of such scholarship in the case of health information management education. The current research contributes to amelioration of this situation.

Our research has revealed complexities in the arrangements that scaffold LTU’s capstone WIL program for its health information management students. The findings have demonstrated: a predominance of the project-based placement model compared to the internship and other models; and a wide range of placement agencies which was, incidentally, reflective of the employment destinations of new graduate HIMs. The study has revealed the agencies’ pre-placement expectations of students (knowledge, technical skills and various soft skills) and the consistency of these requirements with findings from the broader WIL literature, the national HIM Profession-entry Competency Standards, and course curricula. The findings will strengthen the profession’s knowledge base of health information management practical/professional practice education. They will inform health information management educators and students in all university health information management courses, globally, as well as HIM-supervisors in placement agencies. Finally, the findings will scaffold our overarching research study to evaluate the health information management professional practice placement program and identify evidence to inform best practice guidelines for project-based placements.

Our research has demonstrated that LTU has a robust and comprehensive framework for its capstone WIL program for health information management students. Aspects of the current practices, including project-based placements, are shown in the higher education literature to provide substantial post-graduation employment-related benefits for students. The findings reinforce current practices designed to enhance the production of future-ready, graduate HIMs. These include ensuring that future project-based WIL accommodates ILOs and supports students’ consolidation of profession-entry competencies and career-ready soft skills.

## Supplemental Material

sj-docx-1-him-10.1177_18333583241303771 – Supplemental material for Health information management students’ work-integrated learning (professional practice placements): Where do they go and what do they do? Supplemental material, sj-docx-1-him-10.1177_18333583241303771 for Health information management students’ work-integrated learning (professional practice placements): Where do they go and what do they do? by Kerin Robinson, Merilyn Riley, Natasha Prasad and Abbey Nexhip in Health Information Management Journal

sj-docx-2-him-10.1177_18333583241303771 – Supplemental material for Health information management students’ work-integrated learning (professional practice placements): Where do they go and what do they do? Supplemental material, sj-docx-2-him-10.1177_18333583241303771 for Health information management students’ work-integrated learning (professional practice placements): Where do they go and what do they do? by Kerin Robinson, Merilyn Riley, Natasha Prasad and Abbey Nexhip in Health Information Management Journal
