# Supplementary material for: Health information management students’ work-integrated learning (professional practice placements): Where do they go and what do they do?
Source: Health Inf Manag. 2024 Dec 18;54(3):279–89. doi: 10.1177/18333583241303771 (PMC12398634; doi:10.1177/18333583241303771)
Supplement: sj-docx-1-him-10.1177_18333583241303771 – Supplemental material for Health information management students’ work-integrated learning (professional practice placements): Where do they go and what do they do? [file sj-docx-1-him-10.1177_18333583241303771.docx]

**Appendix A: Key definitions**

| **Term** | | **Definition** |
| --- | --- | --- |
| ***Major or Minor Project*** | | |
| Major | A substantive output or body of work (i.e. report, policy and/or procedure manual, data dictionary, mapping document). Substantial audit, e.g., organisation wide, resulting in a change in policy and/or procedure; includes large clinical audits. | |
| Minor | Anything that is not considered a major output is classified as minor; e.g., minor audit or development of educational material where this activity is not the major focus of the placement. | |
| Major or minor | The proposal outlines multiple options in which both major and minor outputs are listed. | |
| Not applicable | Where ‘HIS internship’ or ‘Internship (Other)’ is selected as the placement category. Where the Internship proposal specifies a project, select ‘minor’ project. | |
| Unknown/not specified | Where it cannot be determined whether the project is major or minor. | |
| ***Placement Sub-category*** | | |
| Hospital-based Health Information Service (HIS) internship | Generic HIS activities not specifying any major tasks. Where major tasks are specified, select from other sub-categories. Includes documentation audits (not revenue focused). | |
| Informatics/Health IT | Implementing a new IT system, or the primary focus includes IT systems, e.g., health information/records management systems. Relates to any phase of the system development life cycle. | |
| Epidemiological Research or Medical Research or Clinical Trials | Activities involve a research project or contribution to a study, i.e. literature search, data collection, descriptive analysis or coding of research data. | |
| Quality or Clinical Risk Management | Projects or activities that involve assessing quality and/or clinical risk monitoring, e.g., audits. | |
| Clinical Coding and/or Casemix | Clinical coding and/or related activities (e.g., documentation audits related to coding, revenue or casemix). Does not include analysis of coded data: this should be classified to 'Health Data Analysis and/or reporting'. | |
| HIS Special Project | A project that is focused in the HIS. If selected, provide further details on the nature of project, e.g., medical record destructions project, etc. | |
| Disease or Screening Registry or Clinical Database Management | The placement is based in a clinical registry or research institute and involves activities in the management of databases. | |
| Health Data Analysis and/or reporting | Activities that involve using health data, such as extracting, managing, analysing, manipulating and/or reporting. | |
| Mixed options | If selected, provide further details of the varied activities of the placement. | |
| State or national information infrastructure and governance | Activities in organisations that are responsible for the rules, oversight and monitoring of health-related information infrastructures. | |
| Process and/or procedure development or monitoring | For example, a coding-related quality activity **can** fall under process and/or procedure development or monitoring. | |
| Not specified |  | |
