# Supplementary figures and images for: Health information management students’ work-integrated learning (professional practice placements): Where do they go and what do they do?
Source: Health Inf Manag. 2024 Dec 18;54(3):279–89. doi: 10.1177/18333583241303771 (PMC12398634; doi:10.1177/18333583241303771)

**Appendix B: Internal Rules**


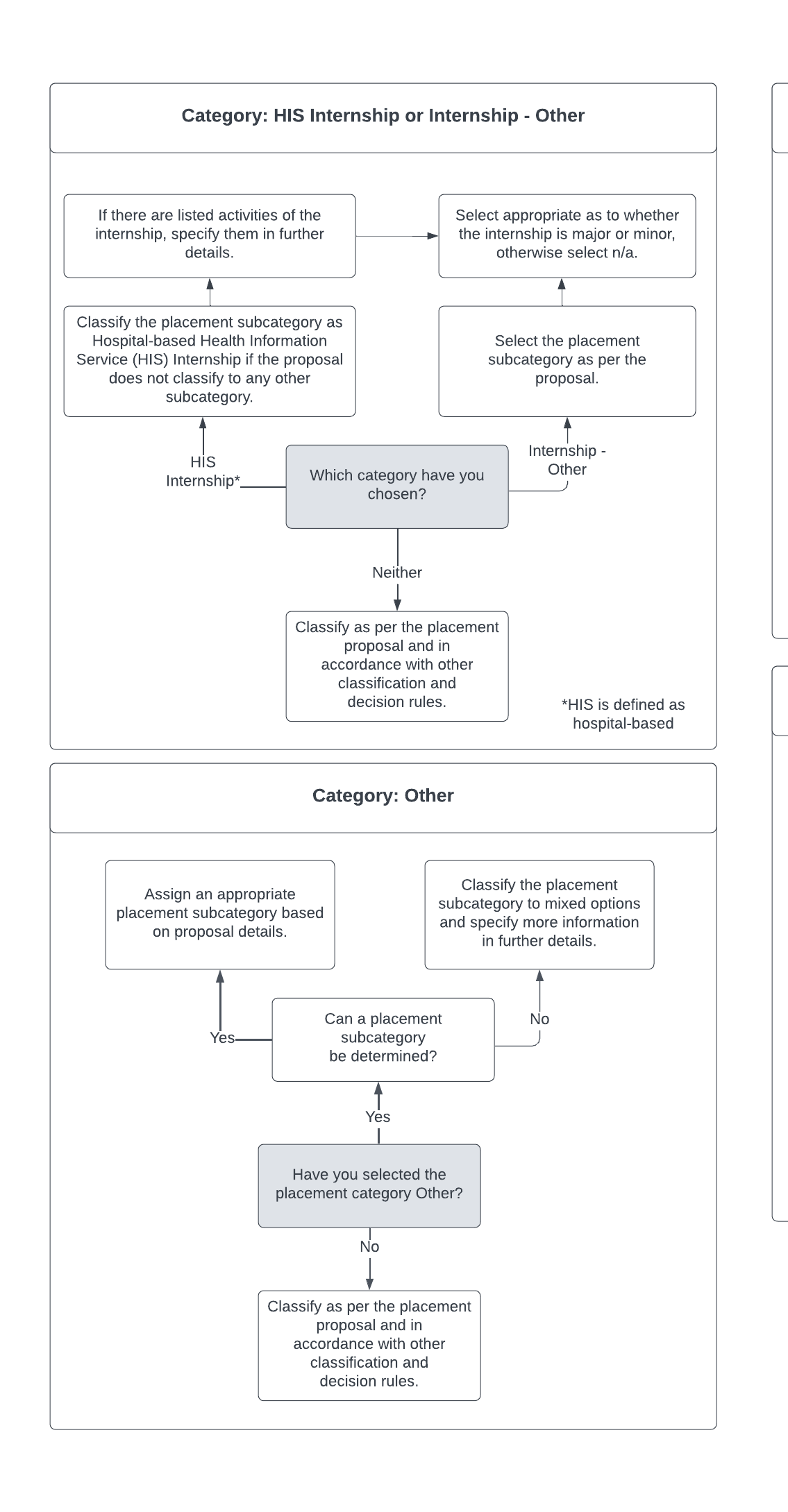


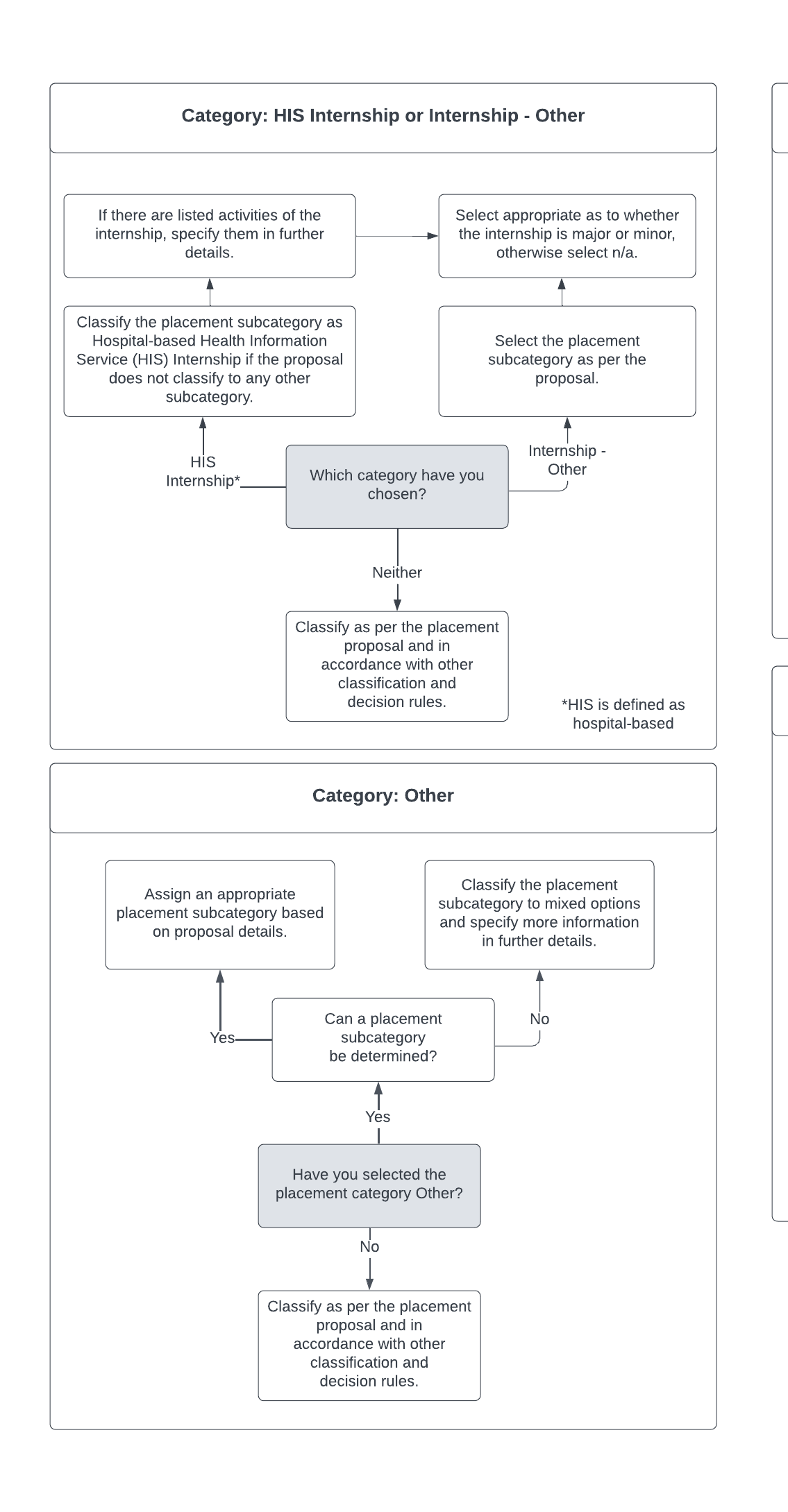


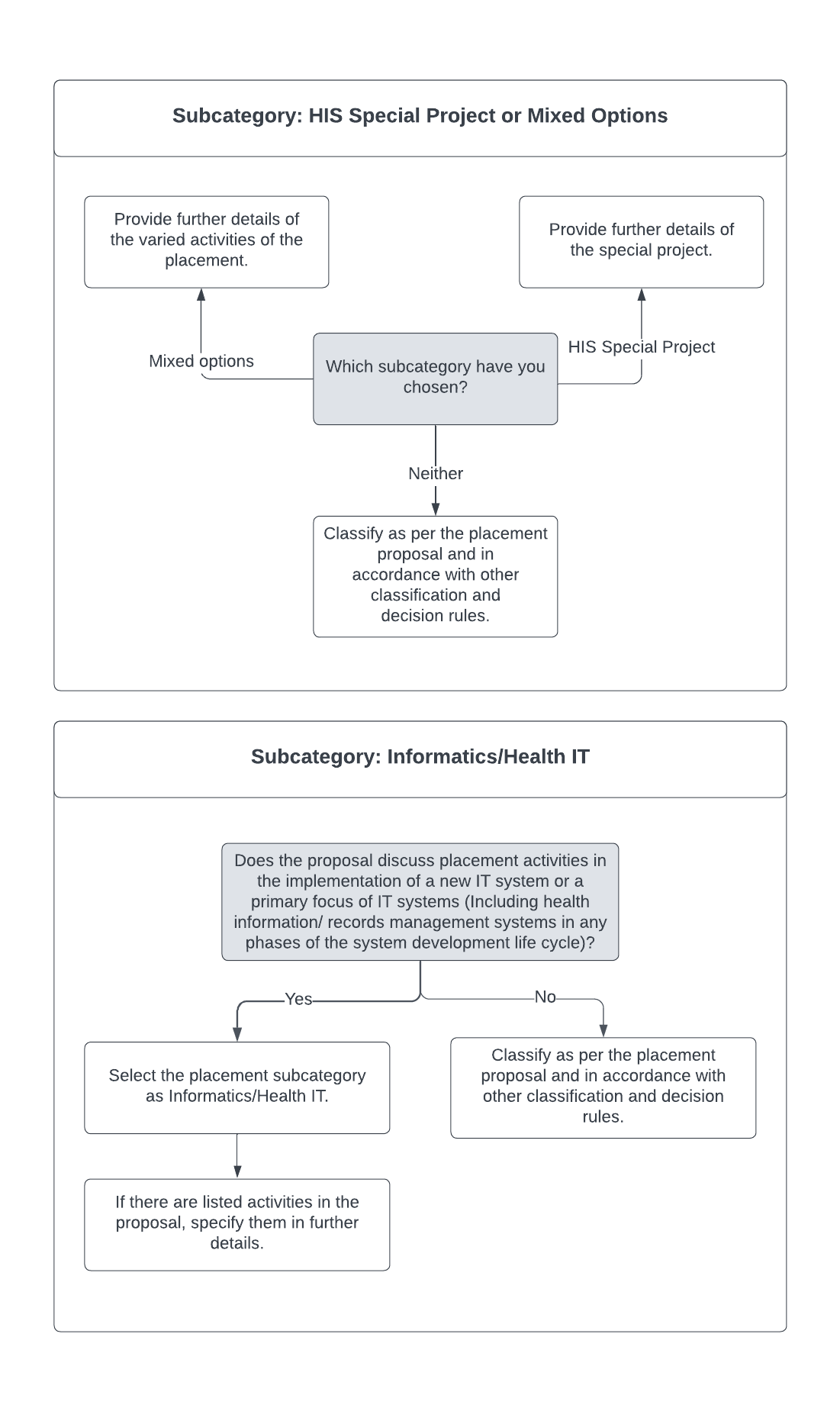


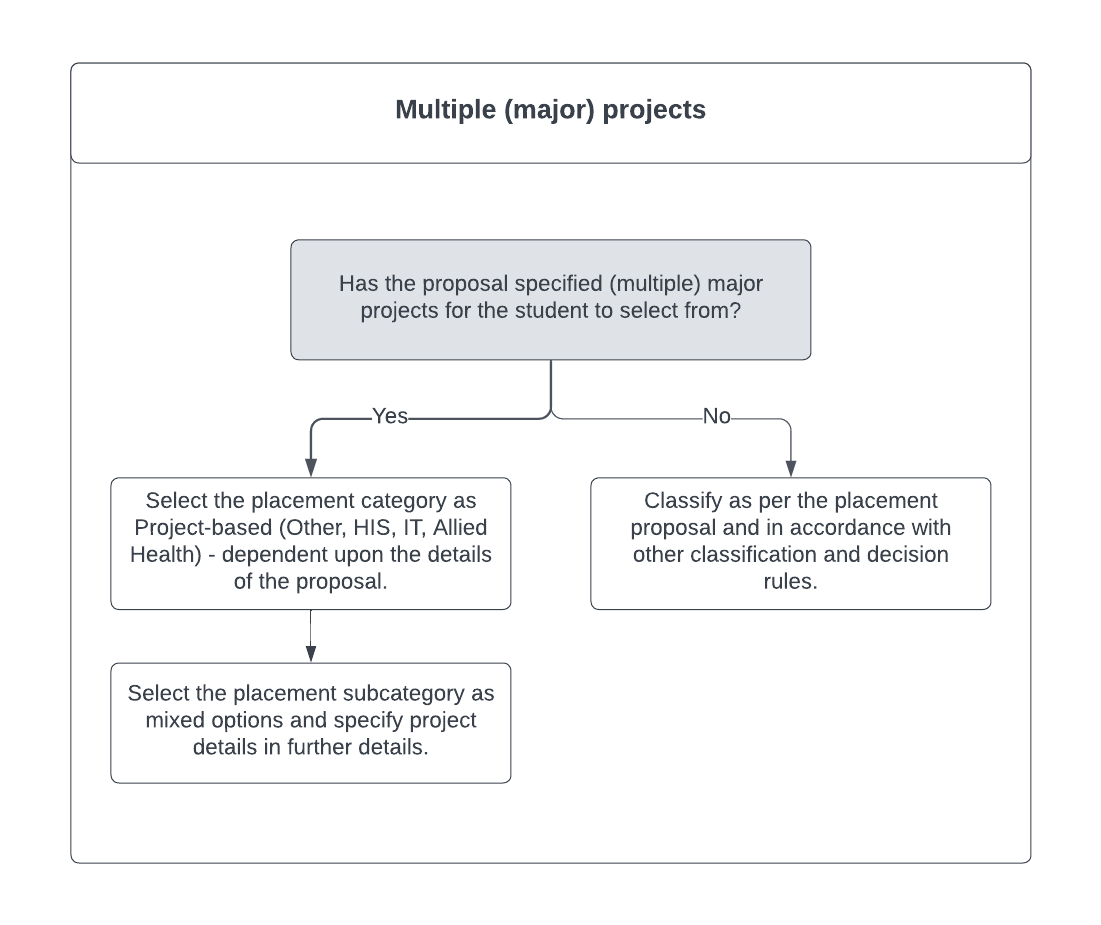

Supplement: sj-docx-2-him-10.1177_18333583241303771 – Supplemental material for Health information management students’ work-integrated learning (professional practice placements): Where do they go and what do they do? [file sj-docx-2-him-10.1177_18333583241303771.docx]
